# Integrating end‐user preferences into breeding programmes for roots, tubers and bananas

**DOI:** 10.1111/ijfs.14911

**Published:** 2021-03-03

**Authors:** Dominique Dufour, Clair Hershey, Bruce R. Hamaker, Jim Lorenzen

**Affiliations:** ^1^ CIRAD UMR Qualisud Montpellier F‐34398 France; ^2^ Qualisud CIRAD Institut Agro Univ Montpellier Avignon Université Université de La Réunion Montpellier France; ^3^ International Consultant Flinton PA USA; ^4^ Whistler Center for Carbohydrate Research Purdue University West Lafayette IN USA; ^5^ Bill & Melinda Gates Foundation Discovery/Crop R&D Global Growth & Opportunity Agriculture Seattle WA USA

## Abstract

“*Consumers have their say: assessing preferred quality traits of roots, tubers and cooking bananas, and implications for breeding*” special issue, brings together new knowledge about quality traits required for roots, tubers and bananas (RTB) varieties to successfully meet diverse user preferences and expectations, along the variety development and RTB value chains (production, processing, marketing, food preparation, consumption). Key RTB crops in sub‐Saharan Africa are cassava, yams, sweetpotatoes, potatoes and bananas/plantains. They are mainly consumed directly as boiled pieces or pounded in the form of smooth, not sticky, and stretchable dough. They are also stewed, steamed or fried. Cassava, the most widely grown RTB, is generally boiled, stewed or steamed in Eastern and Southern Africa, and in West and Central Africa is usually processed directly into derivative products, e.g. whole root fermentation through retting or heap fermentation; fermentation/dewatering of the mash. Biophysical and social knowledge presented in this issue help elaborate goals for both the processing unit operations (food scientist control) and variety traits (breeder control).

In this article, we introduce the key root, tuber and banana (RTB) crops and their derived food products in sub‐Saharan Africa (SSA), summarise the goals and methodologies of assessing end‐user preferences aimed at breeding suitable varieties and highlight some key findings of the research reported in the special issue – *Consumers have their say: assessing preferred quality traits of roots, tubers and cooking bananas, and implications for breeding*. This issue includes 23 original research papers, five review papers and one methodology paper. The authors are affiliated with national research programmes, universities, CGIAR centres (IITA (Nigeria), CIP (Peru) and the Alliance of Bioversity International and CIAT (Colombia)), NRI, CIRAD, NGOs and others. The core contributors present results from the RTBfoods project (*Breeding roots tubers and banana products for end‐user preferences* https://rtbfoods.cirad.fr/ ) and/or the CGIAR Research Program on Roots, Tubers and Bananas https://www.rtb.cgiar.org/. The Bill & Melinda Gates Foundation https://www.gatesfoundation.org/ supports these developments by investing in strategic RTB‐related initiatives in SSA. A call for papers by the journal editors elicited an additional six original research papers.

Following a consultation with RTB experts from different backgrounds and disciplines, 11 food products particularly important for RTB‐based staple diets in SSA were selected for the research reported in this issue. Multidisciplinary teams of breeders, social scientists, food technologists and others used new methods for capturing user quality preferences (Forsythe *et al*., 2021; Teeken *et al*., 2021), through surveys and product evaluations conducted with farmers, traders, processors and consumers. It is notable that the Forsythe *et al*.'s paper (described further below) is the first registered report to be published by IJFST.

The original research papers bring together new knowledge about quality traits required for RTB varieties to successfully meet diverse user preferences and expectations, at each step along the variety development and RTB value chains (production, processing, marketing, food preparation, consumption). Papers address key problems in the breeding of RTB crops in SSA: inadequate understanding of requirements for different end uses, missing information on the physicochemical factors determining these requirements and absence of high‐throughput screening protocols. The review papers provide a broad overview of RTB production and use trends (Scott, 2021), describe case studies of varietal change (replacing old varieties with new ones) (Thiele *et al*., 2021), summarise analytical methods for rapid quality assessment of yam and cassava using near‐infrared spectroscopy (Alamu *et al*., 2021), describe gari end‐user preferences (Awoyale *et al*., 2021) and review the literature to develop a product profile for fried sweet potato in West Africa (Carey *et al*., 2021).

Roots, tubers and bananas play an essential role as staple foods in the tropics and subtropics, and particularly in Africa where they are culturally highly valued (Muimba‐Kankolongo, 2018; Kennedy *et al*., 2019; Lebot, 2019; Petsakos *et al*., 2019; Scott, 2021). Key RTB crops in the African context are cassava, cooking bananas/plantains, yams, sweet potatoes and potatoes (FAO, 1990; Lebot, 2019). Due to their adaptability in different ecosystems and high yields compared with local cereals, they are a primary and reliable source of calories for populations in SSA. However, these crops are highly perishable and difficult to store or transport due to their bulkiness and high moisture content. In order to overcome these issues, and in the case of cassava, to reduce levels of the toxic cyanogenic compounds, a wide range of processes have been developed over millennia to convert fresh roots and tubers into a wide array of more stable and safe products with preferred local traits (Hahn, 1989).

Table 1, based on FAO's *new food balance* data FAOSTAT, (2018), shows the overwhelming dietary importance of cassava, yam and cooking bananas in SSA, with total RTB consumption well above 100 kg per capita per year in most countries. By way of comparison, RTB consumption in Europe and North America is around 60 kg per capita per year, mainly potatoes.

**Table 1:**
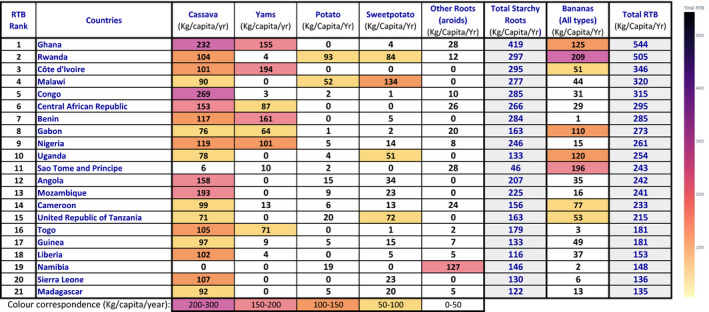
RTB food supply ranking in principal producing countries of Africa (2017) (kg per capita per year)

Faostat, new food balance. http://www.fao.org/faostat/en/#data/FBS

Faostat, new food balance. http://www.fao.org/faostat/en/#data/FBS

The ranking of countries (first column) corresponds to the importance of total RTBs in local diets, as shown in the last column. FAO estimates that 342 million tonnes of RTBs were produced in Africa in 2018, 34% of which in Nigeria alone and 52% in West Africa (FAOSTAT, 2018). Cassava contribute 50%; yam, 21%; sweet potato, 8%; potato, 8%; cooking bananas, 6%; and dessert bananas, 4% of the African RTB production (FAOSTAT, 2018; Lescot, 2020). This major RTB consumption is distributed across the SSA tropical belt (Fig. 1).

**Figure 1:**
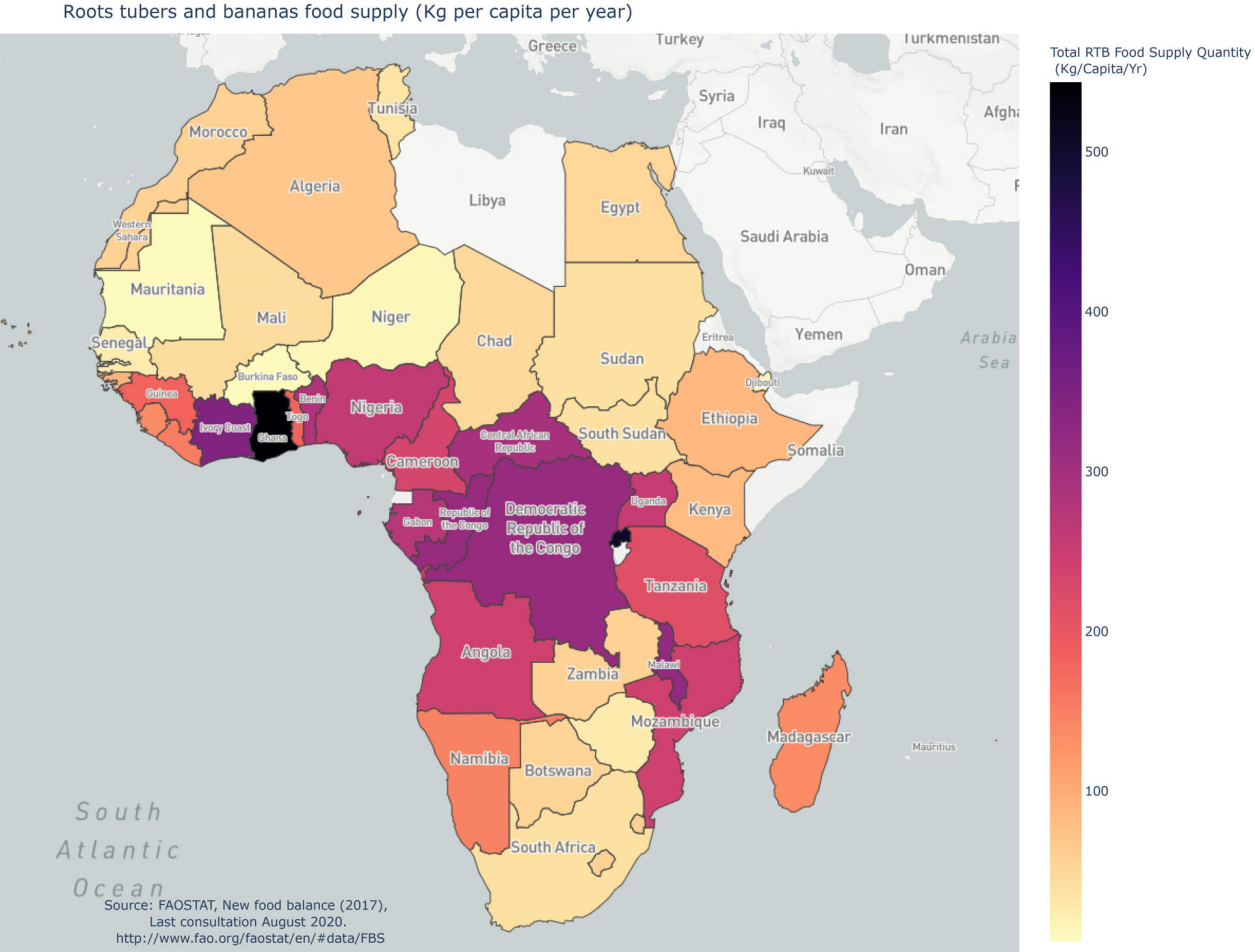
Map of RTB food supply in Africa, 2017 (kg per capita per year).

Cassava, yam and plantain contribute to the development of original African cuisines without mimicking exogenous cuisine or the westernisation of African urban consumption. Bricas *et al*. (2016) showed the capacity of African urban markets to drive the development of RTB local food chains in relation to the preferences and needs of users. A research priority is to adapt RTB value chains to this urbanising context so that consumers can have ready access to these foods and more nutritious options (Petsakos *et al*., 2019; Thiele & Friedmann, 2020).

In Africa, women play crucial roles in the production, processing and preparation of RTB crops, but their input is often under‐valued in technology design that may affect any of these activities (Rubin & Manfre, 2014; Weltzien *et al*., 2020). Many of the studies in this special issue looked specifically at the current and potential women's benefits through their participation in optimised product quality evaluation and critical feedback to breeding programmes.

RTBs are mainly consumed directly in SSA as boiled pieces or pounded in the form of smooth, not sticky, and stretchable dough. They are also stewed, steamed or fried and eaten with African traditional soups. Cassava is generally boiled, stewed or steamed in eastern and southern Africa, and in West Africa is usually processed directly after harvest into derivative products, for example whole root fermentation and softening through retting (fufu, lafun, bâton, chikwangue); heap fermentation (kwon); and fermentation/dewatering of the mash after grinding, followed by steam cooking (attiéké), roasting (gari) and dough formation (eba). All these unit operations are part of the formulation of food products with quality traits strongly sought after by local consumers. Hahn (1989) showed the importance of these different processing techniques in relation to the geographical distribution of key cassava products in SSA. Farmer processors and consumers have strong preferences in terms of raw material quality, depending on the processes developed for the formulation of each cassava‐based food (Béchoff *et al*., 2018).

Breeding programmes for RTB crops initially gave priority to yield, dry matter and disease/pest resistance. As progress in these basic requirements has been met, they must now focus on end‐product quality traits and processor and consumer preferences for quality characteristics. Processing ability and quality of end products are a common issue across improved varieties of RTBs, and this can contribute to low levels of varietal adoption and its subsequent benefits (Thiele *et al*., 2021).

To address these challenges, an interdisciplinary five‐step methodology was developed for the RTBfoods project to better understand consumer preferences for quality characteristics among diverse user groups along the food chain (Forsythe *et al*., 2021). The methodology includes a state of knowledge review, consultations with key informants and rural communities, diagnostics of process performance (technical and socio‐economic) with experienced processors, and consumer testing in urban and rural areas. Quality characteristics are then prioritised into a food product profile. Through testing and analysis, individual quality characteristics are associated with biochemical and physical traits of RTB fresh materials, which can be shared with breeders to develop improved selection tools. Many of the papers in the special issue report result from the application of this methodology. Physicochemical scientists, food technologists and breeders then need to jointly develop and implement high‐throughput phenotyping systems to screen large numbers of potentially improved genotypes and achieve significant genetic gains towards acceptable varieties for both producers and end users.

According to research reported in this special issue, the main characteristics preferred by RTB consumers of boiled products are rapid softening and/or the development of friability or mealiness during cooking. For pounded products, the main objective is to obtain a smooth and stretchy dough with easy‐to‐swallow texture, colours and taste/aroma typical of each RTB (matooke, pounded yam, eba). For cassava‐derived products, colour, acidity due to fermentation, stickiness in the fingers and texture of the dough in the mouth are key sensory traits. Additionally, the swelling characteristics of gari (in cold or hot water), attiéké (during steaming) and fufu (in hot water) contribute to consumer preferences.

In summary, this special issue defines and communicates an improved knowledge of the end‐user preferred quality characteristics of RTB products. This knowledge helps elaborate goals for both the processing unit operations (food scientist control) and variety traits (breeder control). A dynamic and close interaction among social scientists, food technologists, biochemists and plant breeders will contribute to better end‐user acceptance and the successful deployment of technologies, for a positive impact on RTB value chains in Africa.

## Author Contributions

**Dominique Dufour:** Conceptualization (lead); Funding acquisition (lead); Investigation (supporting); Methodology (supporting); Project administration (lead); Supervision (lead); Validation (equal); Writing‐original draft (lead). **Clair H Hershey:** Conceptualization (supporting); Writing‐original draft (supporting); Writing‐review & editing (lead). **Bruce Rankin Hamaker:** Conceptualization (equal); Writing‐review & editing (equal). **Jim Lorenzen:** Conceptualization (equal); Funding acquisition (lead); Project administration (supporting); Resources (lead); Supervision (supporting); Validation (supporting).
